# 气管支架置入患者的预后相关因素分析

**DOI:** 10.3779/j.issn.1009-3419.2020.104.04

**Published:** 2020-06-20

**Authors:** 越群 牛, 沙 黄, 舟 安, 杰 汤, 望 吕, 坚 胡

**Affiliations:** 310003 杭州，浙江大学医学院附属第一医院胸外科 Department of Thoracic Surgery, the First Affiliated Hospital, School of Medicine, Zhejiang University, Hangzhou 310003, China

**Keywords:** 气管支架, Charlson合并症指数, 操作时间, 预后, Airway stent, Charlson comorbidity index, Procedure duration, Prognosis

## Abstract

**背景与目的:**

气管支架被用于治疗各种良恶性气管疾病以及医源性操作引起的中央气道狭窄或气管瘘，其在迅速缓解症状方面具有较好的效果，但患者的长期生存仍有赖于对原发病的个体化治疗。因此，探索影响气管支架置入患者预后的相关危险因素，对于优化支架置入术以及改善患者的个体化临床管理具有帮助作用。

**方法:**

回顾性研究2014年1月-2017年6月在浙江大学附属第一医院胸外科接受支气管镜下气管支架置入治疗的66例患者，分析年龄、性别、基础Charlson合并症指数（Charlson comorbidity index, CCI）、支架植入操作时长等临床指标对患者预后的影响。

**结果:**

年龄、性别对患者预后没有显著影响，而CCI评分较高（*P*=0.045）和操作时间超过60 min（*P*=0.037）均为患者预后不良的独立危险因素，随后依据CCI评分和操作时长两个指标构建了患者预后的列线图预测模型，模型的受试者工作特征曲线（receiver operating characteristic curve, ROC）的曲线下面积为0.71，一致性指数为0.69。

**结论:**

对于接受气管支架置入的患者，其基础的CCI评分和支架置入操作的时长对其预后判断具有一定的临床价值。

## Abstract (en)

**Background and objective:**

Airway stents are used to treat central airway stenosis or tracheal fistula caused by a variety of malignant and benign tracheal diseases as well as iatrogenic procedures. Airway stent placement has a satisfying effect in instantly relieving of symptoms, but the long-term survival of patients still depends on the individualized treatment of the primary diseases. Therefore, exploring the prognostic risk factors of patients who received airway stent placement can be beneficial to the optimization of the placement procedure and also the improvement of individualized clinical management of patients.

**Methods:**

Data of a total of 66 patients who underwent airway stent placement at the First Affiliated Hospital of Zhejiang University from January 2014 to June 2017 were retrospectively collected. Prognostic effects of the clinical characteristics as age, gender, Charlson comorbidity index (CCI) and procedure duration were analyzed.

**Results:**

Age and gender had no significant effect on the outcomes of the patients, while higher CCI (*P*=0.045) and procedure duration over 60 min (*P*=0.037) were both independent risk factors of poor prognosis. A prognostic nomogram was then constructed, of which the area under the curve of the receiver operating characteristic (ROC) curve and the concordance index (C-index) was 0.71 and 0.69, respectively.

**Conclusion:**

For patients receiving airway stent placement, the baseline CCI and the procedure duration had prognostic significance in clinical practice.

以肺癌和食管癌为首的许多恶性疾病以及部分气道良性疾病可引发各类气道病变，例如中央气道梗阻、气管食管瘘等，并产生呼吸困难、阻塞性肺炎等并发症，病情危重者可能因进行性呼吸困难而产生窒息的风险，严重威胁患者的生命健康。而气管支架置入术常被视为快速缓解中央气道狭窄和维持气管完整性的重要手段，具有令人满意的效果^[1]^。此外，对于部分恶性肿瘤终末期患者，使用气管支架来治疗继发于肿瘤的气管食管瘘，提高患者生活质量，也是一种较好的选择^[2]^。然而，虽然气管支架置入术对于缓解中央气道狭窄患者的临床症状效果较好，但伴随的支架相关并发症并不少见，主要包括支架移位（5%-20%）、肉芽组织增生（15%-20%）、痰栓形成（10%-20%）、支架断裂（10%）等，其他还包括肺不张、再狭窄以及感染等诸多并发症^[3, 4]^；加之患者基础疾病通常较危重，尤其是继发于恶性肿瘤者，确诊时大多已错失手术治疗机会^[5]^，因此接受气管支架置入术治疗的患者预后往往不尽如人意，对这部分患者的预后情况进行深入的分析和研究具有重要的临床意义。在此我们回顾性地分析了本中心近年来接受气管支架置入术患者的临床资料和预后状况，旨在探索对预测此类患者预后具有重要意义的临床指标，并进一步构建预后预测模型，为气管支架置入患者的临床管理和决策提供新的参考。

## 1 资料与方法

### 1.1 患者资料

选取2014年1月-2017年6月在浙江大学附属第一医院胸外科接受气管支架置入治疗的患者66例。所有患者无气管支架置入术的操作禁忌证，本研究方案经浙江大学医学院附属第一医院伦理委员会批准。患者术前的Charlson合并症指数（Charlson comorbidity index, CCI）被采纳作为评估患者基础身体健康状况的指标之一；所有患者在行气管支架置入治疗后症状均有所缓解，并采用了Karnofsky行为表现量表（Karnofsky performance status, KPS）对患者接受气管支架置入治疗前和治疗后的身体功能状况进行了评价。

### 1.2 气管支架置入方法

患者取局麻或全麻后，常规消毒支气管镜、置入器，术者首先通过支气管镜检查确认病变所在位置，使用硬性支气管镜（Karl-Storz，德国）时需要患者全身麻醉，使用软性支气管镜（Olympus，日本）时则采用2 mL-3 mL的2%利多卡因进行局部麻醉。当病变位置及使用的支架型号确定后，将导丝送入病变部位远端，留置导丝退出纤支镜，经导丝引导下将带有气管支架的推送器送入并将支架对准病变部位后，迅速回抽推送器导鞘即可释放气管支架，然后退出置入器及导丝，经纤支镜检查确认支架形态、撑开情况并处于合适的位置后可拔出纤支镜。置入后24 h进行首次支气管镜随访检查。

### 1.3 统计方法

总体生存率是本研究的主要结果。计量资料用均数±标准差（Mean±SD）表示，对于支架置入前后的KPS评分比较采用配对*t*检验；计数资料以率（%）表示；使用*Kaplan-Meier*分析和对数秩（*Log-rank*）检验比较不同患者群体间总体生存率的差异；使用单因素和多因素的*Cox*比例风险回归模型比较不同检测指标对于患者预后的预测能力，其中年龄和CCI评分为连续变量，性别和操作时间（≤60 min*vs* > 60 min）以分类变量的形式纳入分析，患者死亡的风险比（hazard ratio, HR）及其95%置信区间（confidence interval, CI）被用于评价每个指标对预后的具体影响；列线图（nomogram）法^[6]^被用于气管支架置入术患者预后预测模型的构建；受试者工作特征曲线（receiver operating characteristic curve, ROC）及ROC曲线的曲线下面积（area under the curve, AUC）被用于对预测模型进行评价；采用一致性指数（index of concordance, C-index）对预测模型与真实值之间的区分度，即预测模型的预测精度进行评价，不同模型间C-index的比较采用Z检验的方法。所有数据分析通过SPSS 19.0和R 3.5.2完成。*P* < 0.05时认为具有统计学显著性差异。

## 2 结果

### 2.1 一般情况

在66例接受气管支架置入术治疗的患者中，男性50例（75.8%），女性16例（24.2%）；年龄26岁-90岁[平均年龄（63.45±10.85）岁，中位年龄63岁]；基础疾病方面，肺癌患者29例（43.9%），食管癌患者27例（40.9%），其他（包括甲状腺肿块、纵隔肿瘤、气管切开后狭窄、黏膜炎等）患者10例（15.2%）；治疗目的方面，因气道新生物引起狭窄者36例（54.5%），外源性压迫引起狭窄者10例（15.2%），气管食管瘘患者19例（28.8%），外源性压迫狭窄并发瘘道形成者1例（1.5%）；术前Charlson合并症指数0分-11分，平均（5.4±2.6）分，中位得分5分；支架置入操作总时长5 min-177 min，平均（44.7±35.5）min，中位操作时长32.5 min。对其中27例患者气管支架置入前后的KPS评分进行统计分析，发现气管支架置入治疗显著提高了患者的KPS评分[（56.3±22.6）分*vs*（81.1±19.1）分，*P* < 0.001）]。

### 2.2 气管支架置入患者预后风险因素分析

通过*Kaplan-Meier*生存分析，我们发现术前CCI评分高于5分和操作时间超过60 min的患者预后相对较差。结果显示，术前CCI评分较低（≤5分）的患者预后显著优于CCI评分高（> 5分）的患者（*P*=0.045）（图 1A）；而操作时间短（≤60 min）的患者预后显著优于操作时间长（> 60 min）的患者（*P*=0.037）（图 1B）。这些结果表明，术前CCI评分和操作时长对于气管支架置入患者的预后具有一定的预测意义。

**1:**
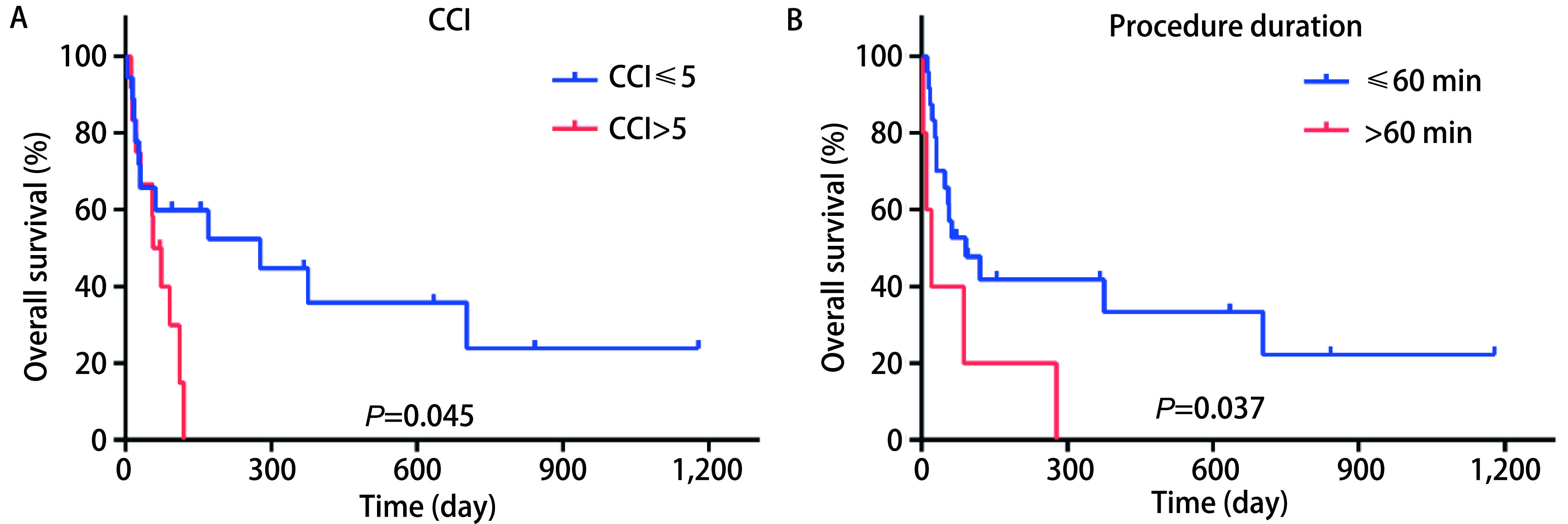
生存曲线。基础CCI评分（A）与操作时间（B）对气管支架置入患者总体生存率的影响。 Survival plots. The effects of baseline CCI (A) and procedure duration (B) on the overall survival of patients receiving airway stent placement.

接下来我们对年龄、性别、术前CCI评分和操作时长进行了单因素*Cox*回归分析，发现患者气管支架置入术前CCI评分高（HR=1.266, 95%CI: 1.014-1.581, *P*=0.037）和操作时间超过60 min（≤60 min *vs* > 60 min, HR=0.350, 95%CI: 0.124-0.985, *P*=0.047）均为预后不良的显著风险因子，而年龄与性别未体现出对预后的显著影响（表 1）。

**1:** 单因素*Cox*分析气管支架置入预后风险因子 Univariate *Cox* regression analysis for airway stent placement

Risk factor	HR	95%CI	*P*
Age	0.987	0.939-1.038	0.610
Gender (Female *vs* Male)	0.600	0.178-2.030	0.412
CCI score	1.266	1.014-1.581	0.037
Procedure duration (≤60 min *vs* > 60 min)	0.350	0.124-0.985	0.047
CCI: Charlson comorbidity index.			

我们进一步对年龄、性别、术前CCI评分和操作时长进行多因素*Cox*回归分析，发现术前CCI评分高（HR=1.561, 95%CI: 1.090-2.238, *P*=0.015）、操作时间超过60 min（≤60 min*vs* > 60 min, HR=0.127, 95%CI: 0.023-0.703, *P*=0.018）均为接受气管支架置入治疗的患者预后不良的独立危险因素，而年龄与性别同样未体现出对患者预后的显著影响（表 2）。

**2:** 多因素*Cox*分析气管支架置入预后风险因子 Multivariate *Cox* regression analysis for airway stent placement

Risk factor	HR	95%CI	*P*
Age	1.001	0.945-1.061	0.960
Gender (Female *vs* Male)	1.030	0.267-3.967	0.966
CCI score	1.561	1.090-2.238	0.015
Procedure duration (≤60 min *vs*. > 60 min)	0.127	0.023-0.703	0.018

### 2.3 气管支架置入患者预后预测模型的建立

根据上述结果，我们同时纳入术前CCI评分和操作时长这2个指标，采用列线图的方法构建气管支架置入患者的预后预测模型（图 2）。为进一步验证此预测模型的有效性，我们绘出了模型的ROC曲线，并计算其曲线下面积AUC=0.71；若采用同样的方法构建由年龄、性别2个指标组成的预后模型，其ROC曲线的曲线下面积只有0.47，两模型ROC曲线对比见图 3。此外，C-index被认为对模型的预测能力有较好的评价功效，我们构建的由CCI评分和操作时长组成的预测模型C-index=0.69（*P*=0.013），而由年龄、性别组成的预测模型C-index仅为0.61（*P*=0.131），两者具有显著差异（*P*=0.046）。因此，我们认为此预测模型对于气管支架置入患者的预后是具有一定预测能力的。

**2:**
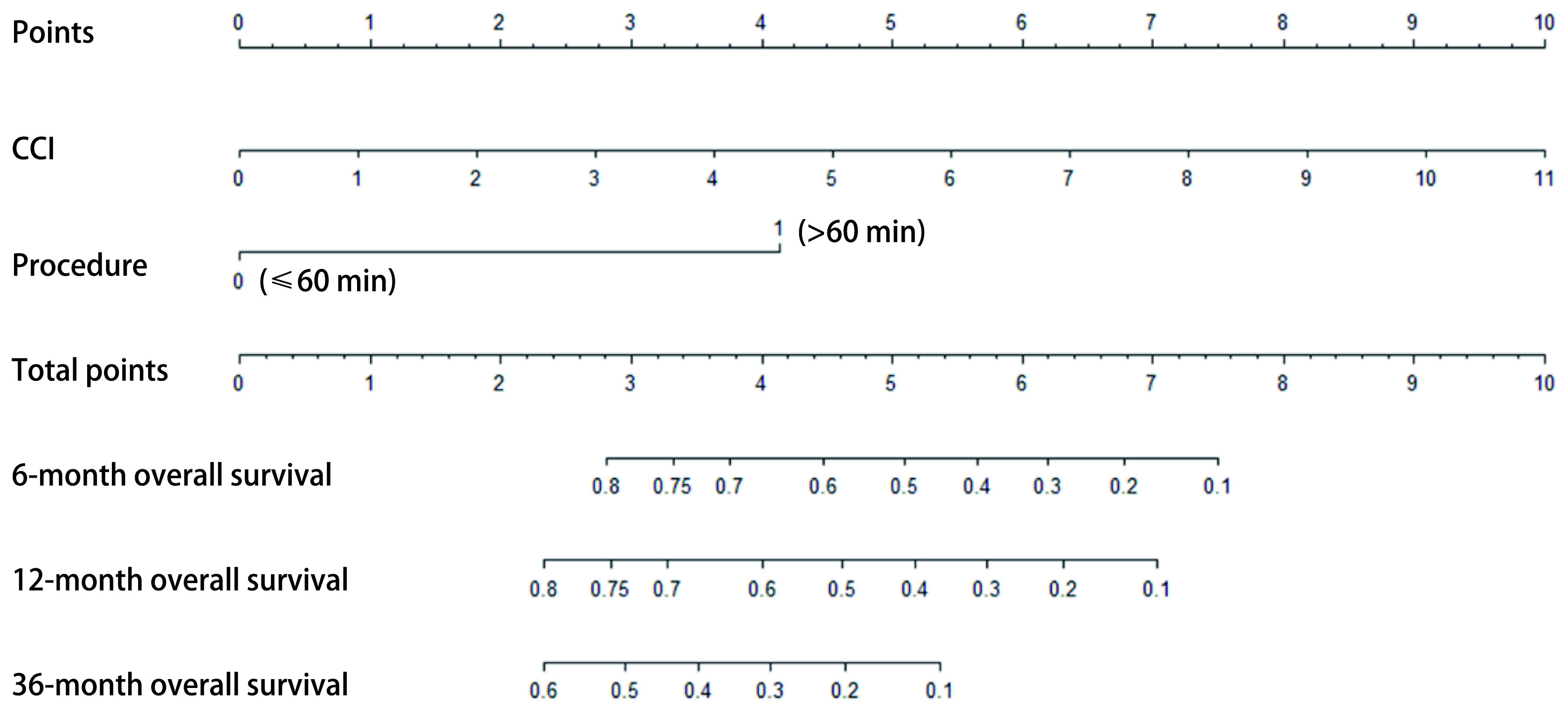
气管支架置入患者预后的列线图预测模型 Prognostic nomogram for prediction of overall survival of patients receiving airway stent placement.

**3:**
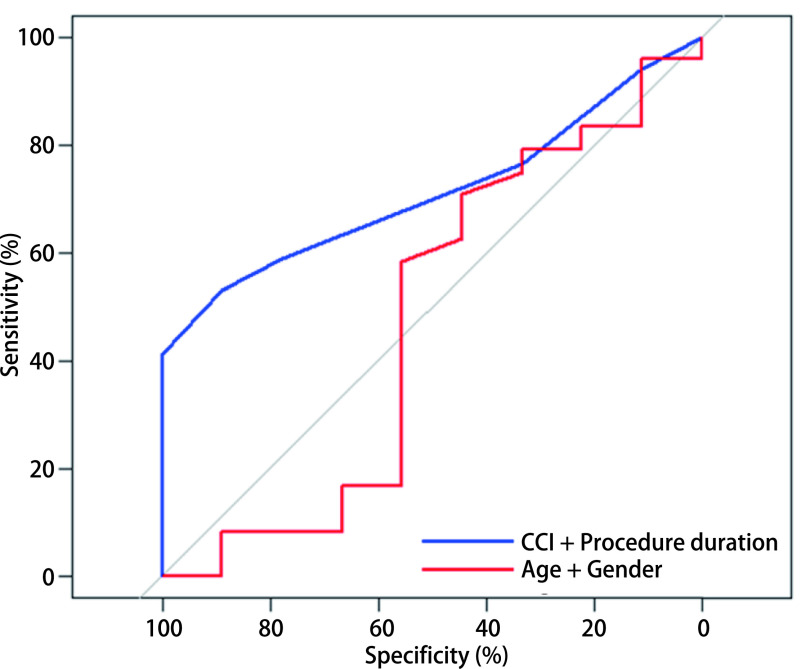
由CCI和操作时间构成的预测模型与由年龄和性别构成的预测模型的ROC曲线对比 Comparison between the prediction model of CCI plus procedure duration and the prediction model of age plus gender. ROC: receiver operating characteristic curve.

## 3 讨论

随着肺癌和食管癌等胸部肿瘤疾病给人类健康带来的负担逐年加重^[7, 8]^，以及人工气道如气管内插管等操作在更广泛和更高龄人群中的操作例数不断增加，继发于良恶性疾病以及气道医源性操作的中央气道狭窄已成为不容小觑的健康威胁之一^[9]^。气管支架置入术可以迅速缓解中央气道狭窄患者的呼吸困难症状，改善患者肺功能，提高患者生活质量，近期疗效较为显著。但支架置入属于姑息性治疗，仅能缓解症状，对疾病进展不具有控制作用^[10]^。因此，关注气管支架置入患者的预后情况，积极调整原发病治疗方案，具有重要的临床意义。

在本研究中，我们对因肺癌、食管癌所致气道病变、气道良性疾病和医源性操作等而接受气管支架置入的患者进行了预后风险因素的分析，患者接受支架置入后症状均有明显改善，患者的KPS评分在支架置入后显著提高，表明气管支架置入对于缓解此类患者症状、提高其生活质量确有重要意义。在进一步的生存分析中，我们发现患者在接受气管支架置入前的CCI评分状态和支架置入术的操作时长对于患者的预后有着重要的预测意义。Charlson合并症指数在1987年由Charlson等^[11]^提出，包含了19种被赋予不同权重的基础合并症。CCI在许多疾病中都已被证实与患者的预后相关，在一项针对非小细胞肺癌患者的大型临床试验^[12]^当中，研究者发现CCI评分较高者预后更差，而年龄并非总体生存率的影响因素。考虑到肺癌是导致患者接受气管支架置入的主要基础疾病之一，这项研究也从侧面佐证了我们的结果。此外，也有研究^[13]^显示对于不能手术而接受射频消融治疗的肺癌患者，CCI评分也是重要的预后独立危险因子。除肺癌外，CCI评分也被证实与食管癌患者的预后和并发症发生相关^[14, 15]^，这些证据均提示了CCI评分在气管支架置入患者中的潜在临床意义，与本研究的结果一致。我们的结果中另一个与患者预后相关的风险因子是操作时间，结果显示超过60 min的操作时间是患者预后的不利因素。在临床上，对操作时间的影响因素较多，包括患者疾病复杂程度、术者操作熟练程度、术中意外情况以及麻醉相关处理等等，一项大型临床研究^[16]^发现气管支架置入成功与否依赖于术者的经验。曾有研究^[17]^探索了在腹股沟疝修补手术中，操作时间与手术效果之间的关系，发现较长的操作时间减少了再次手术率，但增加了感染及其他术后并发症的发生率。因此，关于操作时间对气管支架置入患者预后的具体影响及其原因，仍有待更大规模的研究进一步探索。

自1965年Montgomery首次使用硅酮T形管来治疗患者的气道狭窄^[18]^，1990年Dumon^[19]^取得突破性进展首次真正实现完全的气管腔内支架，到2013年Fuehner等^[20]^首次报道了可生物降解的支架，气管支架在近年来已取得较大的发展^[3, 9, 21]^。尽管80%以上的气道狭窄患者在支架置入后呼吸困难等症状可以立即得到缓解，但支架置入并非对所有患者均可获益，如伴随置入术后的支架相关并发症可能引起危急生命的严重后果，例如严重的出血、支架断裂、气道新生物等，又例如肺塌陷超过2周的患者，置入支架后不仅肺复张较难，而且可能会因支气管的开放而增加感染的风险^[22]^，因此对于支架置入的适应证以及具体支架类型的选择仍应谨慎分析，通过个体化应用来提高支架置入的疗效和并发症的预防^[21]^。本研究对继发于各种基础疾病而接受气管支架置入的患者进行了预后相关因素分析，发现患者CCI评分和支架植入操作时长对患者预后具有重要意义，并依此构建了预后预测模型，为临床上气管支架置入术的优化以及患者的个体化管理和决策提供了新的参考和思路。
